# Functional and Safety Outcomes of Third-Generation Zimmer Biomet G7^®^ Dual Mobility Total Hip Arthroplasty in Femoral Neck Fractures: A Retrospective Cohort Study

**DOI:** 10.3390/jcm14238350

**Published:** 2025-11-24

**Authors:** Zhuowen Geng, Abdul-Samad Asamu, William Aldridge, Aaron Biing Yann Ng

**Affiliations:** 1 Mid Yorks Teaching Hospital Trust, Pinderfields General Hospital, Aberford Road, Wakefield WF1 4DG, UK; wendy.geng@nhs.net (Z.G.); a.samad1@nhs.net (A.-S.A.); 2 Sheffield Teaching Hospitals NHS Trust, Royal Hallamshire Hospital, Sheffield S10 2JF, UK; william.aldridge5@nhs.net

**Keywords:** femoral neck fractures, dual mobility total hip arthroplasty, prosthesis dislocation, third-generation dual mobility cup, periprosthetic fracture, cemented femoral stem, Oxford Hip Score, elderly trauma

## Abstract

**Background:** Femoral neck fractures (FNFs) in the ageing population carry high risks of postoperative dislocation, with traditional total hip arthroplasty (THA) reporting rates up to 10%. Dual mobility THA (DM-THA) may provide enhanced stability, but evidence for third-generation implants like the Zimmer Biomet G7^®^ system remains limited. **Methods:** This retrospective cohort study evaluated 120 patients (mean age 71.6 years; 74% female) with acute displaced intracapsular FNF treated with DM-THA (2021–2023) using the G7^®^ system. Demographics, surgical details (cemented/uncemented stems), complications, and functional outcomes (Oxford Hip Score (OHS) at one year) were analysed against national benchmarks. **Results:** Zero dislocations and two peri-prosthetic fracture (0.8%, cemented stem) occurred. Thirty-day mortality was 0.8% (below national averages). Functional recovery was excellent (mean OHS: 41/48; 69% scoring ≥40). Surgical complications were minimal (one deep infection 0.8%). Medical complications (anaemia 6.6%, venous thromboembolism 4.2%) were significantly higher in high ASA groups (III-IV). Radiographs confirmed stable implants without loosening. **Conclusions:** The G7^®^ DM-THA system demonstrates exceptional stability and safety in FNF patients, with no dislocation risk and low peri-prosthetic fracture rates—even with cemented stems. These outcomes support its use in high-risk populations, though comparative studies with conventional THA are needed.

## 1. Introduction

Femoral neck fractures (FNFs) impose a major healthcare burden among the elderly, often due to frailty and osteoporosis leading to falls. For displaced intracapsular FNFs, total hip arthroplasty (THA) is frequently favoured over hemiarthroplasty because it can provide better long-term function and quality of life in active patients [1]. However, this functional advantage comes at the cost of a higher risk of postoperative instability. Conventional single-mobility THA implants have a reported dislocation incidence of around 2–10% in primary elective THAs, and this risk can rise toward double digits in fragility fracture patients [2,3,4,5,6]. Such dislocations after hip arthroplasty contribute significantly to morbidity and mortality in this already vulnerable patient population.

Dual mobility (DM) acetabular components have been developed to mitigate the risk of THA dislocation, particularly in high-risk trauma patients who sustained FNF. These designs incorporate a small femoral head that articulates within a larger polyethylene liner, which in turn articulates against the metal acetabular shell, effectively creating a double articulation with an enlarged head–neck diameter and greater jump distance for improved stability [7,8,9]. Modern third-generation DM systems (e.g., the Zimmer Biomet G7^®^ acetabular system) also feature modular shells and liner options to optimise fixation and orientation for each patient [10]. Clinical studies and registry data indicate that using DM cups in FNF patients significantly reduces postoperative dislocation rates compared with conventional single-bearing THA or hemiarthroplasty—often achieving dislocation incidences below 4% [2,3,5,7]. For example, a recent meta-analysis found that THA with dual mobility components had an odds ratio ~0.26 (≈74% relative risk reduction) for dislocation versus standard THA in hip fracture patients [8]. Accordingly, DM-THA has gained popularity for elderly and high-risk populations—including those with neuromuscular disorders, cognitive impairment, or requiring extreme range of motion—as a method to improve hip stability following THA [11,12].

Beyond clinical advantages, recent research has investigated the economic- and population-level impact of DM-THA. A cost–utility analysis by Montgomery et al. established that dual mobility THA can be cost-effective in treating hip fracture patients below 80 years of age, provided it substantially lowers dislocation rates [13]. Similarly, the 2024 UK National Joint Registry shows that dual mobility implants have mid-term revision rates comparable to standard THA in high-risk patients, supporting their selective use to reduce dislocation risk. [14].

However, the potential benefits of DM designs must be balanced against concerns about peri-prosthetic femoral fractures (PPFs), which remain a serious complication. PPF risk is inherently elevated in revision surgeries and in elderly patients with poor bone quality [15,16]. For instance, a recent meta-analysis identified advanced age (>70 years), osteoporotic bone structure (Dorr type C), rheumatoid arthritis, use of uncemented stem, and revision arthroplasty as significant risk factors for PPF (odds ratios ranging from ~1.7 to 4.2) [17]. The constrained design of DM cups, while improving stability, may change load transfer to the femur, resulting in increasing PPF incidences [15]. A single-centre series of 126 hip revisions reported PPFs in 74% of cases using a DM cup versus 26% with a standard cup, implying a 12-fold higher fracture risk with the DM construct [15]. Though selection bias might play a role and larger studies are needed for confirmation, it highlights the importance of implant choice and technique. Notably, contemporary modular systems like the G7^®^ offer intraoperative flexibility (multiple shell sizes, liner offsets, and cemented vs. press-fit stem options) to achieve optimal implant alignment and secure fixation even in poor bone stock [10].

This study seeks to address these gaps by evaluating a cohort of traumatic FNF patients treated with the G7^®^ dual mobility THA system, with the following three primary objectives: (1) Outcomes: assess the patients’ functional recovery, safety (complication profile), and implant radiographic outcomes achieved; (2) Complication rates: determine the incidence of dislocations, PPFs, and other poster-operative complications using standard criteria; (3) Benchmarking: contextualise the above results against NHFD national benchmarks for hip fracture care, including 30-day mortality rates. By focusing on high-risk patients and stratifying outcomes with uniform definitions, this investigation provides evidence to inform implant selection and surgical protocols for hip fracture populations who sustained traumatic injuries and are at higher risk of dislocation.

## 2. Materials and Methods

This retrospective cohort study evaluated patients undergoing primary THA with the Zimmer Biomet G7^®^ DM system for femoral neck fractures at Mid-Yorkshire Teaching Hospitals NHS Trust between January 2021 and December 2023. The study followed the STROBE guidelines for reporting observational research.

### 2.1. Patient Selection

Inclusion criteria were as follows:

- Sustained an acute, displaced intracapsular femoral neck fracture (Garden classification III or IV) confirmed on anteroposterior and lateral radiographs.
- Injury resulting from low-energy trauma.
- Underwent primary total hip arthroplasty (THA) using the Zimmer Biomet G7^®^ dual mobility acetabular system at our institution.
- Considered high risk* for postoperative instability or poor outcome from hemiarthroplasty, based on multidisciplinary surgical assessment.

*high-risk status was defined clinically as patients with one or more of the following features:

• High pre-injury functional demand or independence.
• Radiographic evidence of pre-existing acetabular wear (contraindicating hemiarthroplasty).
• Functional, neuromuscular, or cognitive factors predisposing to dislocation.
• Anatomic considerations (e.g., abnormal femoral offset or version) increasing instability risk.
• Surgeon’s intraoperative assessment of bone and soft-tissue quality.

Exclusion criteria were as follows:

- Pathological fractures.
- Prior ipsilateral hip arthroplasty/peri-prosthetic femoral fractures, or prior proximal femoral surgery.
- Polytrauma patients requiring staged orthopaedic management.
- Incomplete perioperative or follow-up data.

**Figure 1:**
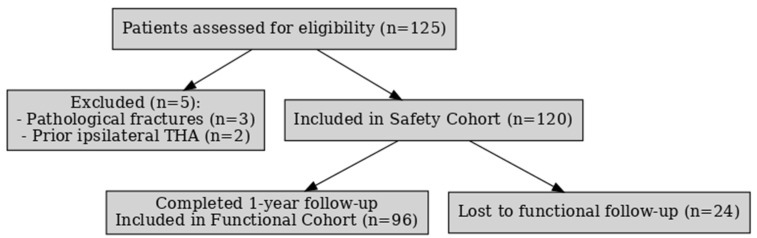
CONSORT-style flowchart.

To mitigate potential selection bias, a two-tiered analytical framework was adopted. The Functional Outcomes Cohort included patients who completed a full 12-month follow-up, incorporating validated Oxford Hip Score (OHS) assessments to evaluate postoperative function. The Safety Cohort comprised the entire population of eligible patients, including those lost to follow-up, to enable comprehensive evaluation of perioperative and postoperative complications. A total of 120 patients met the eligibility criteria of Safety Cohort and 96 in the Functional Cohort. The patient selection process is shown in Figure 1.

### 2.2. Surgical Protocol

All procedures were performed or directly supervised by fellowship-trained orthopaedic consultants. The choice of surgical approach was based on surgeon experience, patient habitus, and fracture configuration.

The posterior approach was employed in most cases, while the anterolateral (Watson–Jones) and direct lateral (Hardinge) approaches were selectively utilised based on patient anatomy, frailty, or surgeon discretion. Patient positioning (lateral decubitus for posterior or direct lateral; supine for anterolateral) was determined by the chosen approach. Due to the study design, exact numbers for each approach were not recorded. Nevertheless, all procedures were performed using a standardised implant system (G7^®^ dual mobility) and uniform perioperative protocols.

Implants used were as follows:

- G7^®^ DM shell;
- Cemented (CPT Hip System Femoral Stem 12/14 Neck Taper—Zimmer Biomet/C-STEM™ AMT—DePutSynthes);
- Uncemented (CORAIL^®^—DePuy Synthes).

Intraoperative details were as follows:

All procedures were performed under either spinal or general anaesthesia. Patients were positioned in the supine or lateral decubitus position, depending on the surgical approach utilised. Acetabular components were implanted using a press-fit technique, with supplemental screw fixation applied at the preference of the operating surgeon. Cemented femoral stems (CPT or C-Stem) were mainly used for patients with compromised bone quality or Dorr type C femoral morphology, whereas uncemented stems (Corail) were employed in cases with adequate metaphyseal support.

### 2.3. Perioperative Management

Perioperative management followed a standardised institutional protocol. Prophylactic antibiotics were administered using 800 mg of intravenous teicoplanin prior to skin incision. Venous thromboembolism (VTE) prophylaxis consisted of both mechanical methods (compression stockings) and chemical prophylaxis with low-molecular-weight heparin, continued for 28 days postoperatively. Patients were mobilised within 24 h of surgery under the supervision of physiotherapists, in accordance with an early mobilisation protocol. All patients received standardised discharge instructions and tailored rehabilitation plans to support recovery.

### 2.4. Outcome Measures

Functional outcomes were as follows:

- Oxford Hip Score (OHS), collected at the routine 12-month in-person clinic visit by a trained orthopaedic surgeon.
- Intra-prosthetic dislocation events.

Safety outcomes were as follows:

- Early (<6 weeks): Surgical site infection (CDC definition), postoperative anaemia**, DVT/PE (confirmed via Doppler or CT-angiography), AKI (KDIGO criteria), or nerve palsy.
- Late (>6 weeks): Peri-prosthetic femoral fracture (PPF)***, dislocation, chronic pain, limb length discrepancy (measured clinically), radiographic loosening, or revision surgery.
  **postoperative anaemia was defined as postoperative Hb < 70 g/L or 7 g/dL and/or requiring transfusion.
  ***Peri-prosthetic fractures (PPFs) were defined as fractures occurring adjacent to the femoral or acetabular components. All PPF events in this series were acetabular in origin; no femoral fractures were observed.

Radiographic analysis were as follows:

Postoperative and 12-month follow-up anteroposterior and lateral pelvic and femoral radiographs were independently reviewed by a senior radiographer and an orthopaedic consultant. Observers were not blinded to clinical details. Radiographic evaluation included assessment for prosthetic dislocation, peri-prosthetic fracture (acetabulum or femoral stem), cup migration and subsidence, and signs of implant loosening.

Acetabular component loosening was assessed using the DeLee and Charnley zonal [18] classification, while femoral component loosening was evaluated based on the Gruen zones [19]. Loosening was defined by the presence of progressive radiolucent lines greater than 2 mm, component migration exceeding 2 mm, or angular tilt of the implant. In instances of interpretative uncertainty or disagreement between the primary reviewers, a second senior clinician was consulted to reach a consensus.

Due to the retrospective design of the study, formal testing for intra- and inter-observer reliability was not undertaken.

### 2.5. Statistical Analysis

All statistical analyses were conducted using Python version 3.12 with Visual Studio Code version 1.101 as the integrated development environment. Descriptive statistics were used to summarise the data. Continuous variables, such as age and Oxford Hip Score (OHS), were reported as means with standard deviations (SDs), while categorical variables were presented as counts and percentages.

The distribution of continuous variables was assessed using the Shapiro–Wilk test to evaluate normality. For non-normally distributed data, the Mann–Whitney U test was applied to compare differences between groups (e.g., OHS between cemented and uncemented stem cohorts). Categorical data with small, expected counts (n < 5) were compared using Fisher’s exact test. A z-test for proportions was used to compare the observed 30-day mortality rate in the study cohort with the 2024 UK National Hip Fracture Database (NHFD) benchmark.

Subgroup analyses were performed to compare clinical outcomes across two key dimensions: fixation method (cemented vs. uncemented stems) and patient comorbidity burden, stratified by ASA classification (≤II vs. ≥III). These comparisons included analysis of both functional outcomes and complication rates.

A two-tailed *p*-value of <0.05 was considered statistically significant. Adjustments for multiple comparisons were not applied given the exploratory nature of the study.

Due to the retrospective design, no a priori power calculation was performed.

### 2.6. Addressing Potential Biases

- Selection Bias: Minimised by inclusion of a complete safety cohort and a separate functional subgroup.
- Measurement Bias: Complications were defined using established clinical criteria. Data extracted independently by two reviewers; discrepancies resolved by a third clinician.
- Observer Bias in Imaging: Radiographic interpretation was consensus-based but lacked formal reproducibility testing.
- Confounding: Frailty, cognitive status and other potential confounding factors were not consistently recorded and are acknowledged as limitations.

### 2.7. Ethical Considerations

This study was conducted as a registered clinical audit at Mid-Yorks Teaching Hospital Trust, and ethical approval was not required under institutional policy for retrospective audits involving anonymised data. Confirmation of this exemption was provided by the institution’s Clinical Audit Department, and the audit certificate was reviewed and accepted by the journal’s editorial board.

## 3. Results

### 3.1. Cohort Demographics

This study included 120 patients who underwent primary total hip arthroplasty (THA) using the Zimmer Biomet G7^®^ dual mobility (DM) system for femoral neck fractures between 2021 and 2023. Of these, 96 patients (80.0%) had completed one-year follow-up and were included in the Functional Cohort. The mean age was 71.6 ± 9.4 years (range: 43–92), with a predominance of female patients (n = 89; 74.2%). Most patients were classified as ASA grade II (61.7%) or III (33.3%), reflecting a high baseline comorbidity burden. Baseline characteristics of the cohort are summarised in Table 1.

### 3.2. Implant Distribution

All patients received the third-generation Zimmer Biomet G7^®^ dual mobility acetabular cup. The distribution of stem types is presented in Table 2. Femoral implant selection was based on intraoperative bone quality and patient comorbidities, with cemented stems more frequently used in older or osteoporotic patients.

### 3.3. Functional Outcomes (n = 96)

**Figure 2:**
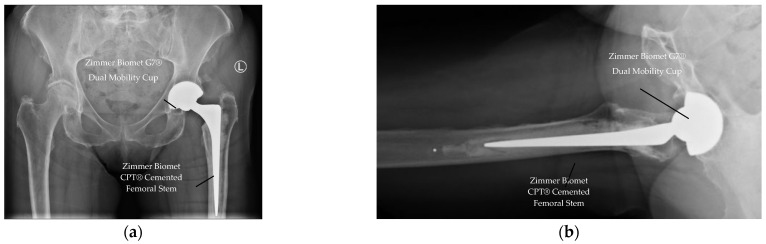
Postoperative radiographs at one-year follow-up. (**a**) Anteroposterior view showing well-positioned G7^®^ acetabular cup and cemented CPT femoral stem (left hip). (**b**) Lateral view confirming absence of radiolucent lines or implant migration.

At one-year follow-up, there was no case of dislocation (0.0%) in any patient (see Table 3), demonstrating excellent stability. All radiographs demonstrated appropriate component positioning of the acetabulum cup and femoral stem, and no radiographic loosening (AP/lateral views) of the implant. See Figure 2 for example of Postoperative radiographs confirmed appropriate component positioning without loosening.

At one-year follow-up, the mean OHS was 41.0 ± 8.3 (range: 5–48), with 68.8% achieving excellent outcomes (OHS ≥ 40). No statistically significant difference in OHS was observed between cemented and uncemented stem groups (*p* = 0.125, Mann–Whitney U test). Detailed OHS values for cemented and uncemented groups are shown in Table 4. 

Radiographic review confirmed no cases of acetabular or femoral component loosening using the DeLee & Charnley (acetabulum) and Gruen zones (femur) classifications. One peri-prosthetic fracture (PPF) of the acetabular implant occurred in the cemented group, resulting in revision at seven months post-initial THA. Periprosthetic fractures at one year are summarised in Table 5.

### 3.4. Safety Outcomes (n = 120)

Complications analyses within the Safety Cohort (n = 120) were stratified to separate early post-op (≤6 weeks) and long-term (>6 weeks) events to identify different targets to assist with recovery at different phases. Overall, no dislocations were observed during follow-up, reinforcing the enhanced stability profile of the G7^®^ dual mobility system.

Early postoperative complications (≤6 weeks) are reported in Table 6. Complications were primarily medical (6.7% Anaemia, 4.2% VTE), rather than implant-related. There was one case of PPF (0.8%), which resulted in revision of the acetabulum. There were no dislocations or early mechanical failures identified at this phase. There were no cases of cup migration, subsidence, or aseptic loosening during the follow-up period. The dual mobility articulation showed no signs of polyethylene wear, dissociation, or intra-prosthetic dislocation.

A comparison of early complications between cemented (n = 93) and uncemented (n = 24) stems is provided in Table 7. It revealed no statistically significant differences in overall or individual complication rates (all *p* > 0.05, Fisher’s Exact test). While numerically higher rates of anaemia were observed in the cemented group (7.5% vs. 4.2%), this difference did not reach significance and should be interpreted cautiously, as no explanation was established. Peri-prosthetic fracture was more frequently noted in the uncemented group than cemented (4.2% vs. 0%). These trends may reflect underlying patient factors or surgical decision-making rather than inherent effects of fixation type.

Due to the absence of early postoperative complications among ASA I and ASA IV patients (n = 0 in these two groups), ASA categories were separated into low-risk (ASA I–II, n = 79) and high-risk (ASA III–IV, n = 41) group for analytical purposes. Stratified early complication rates according to ASA classification are shown in Table 8.

Patients in the high-risk group demonstrated a significantly greater incidences of VTE, including PE/DVT, compared to the low-risk group (9.8% vs. 1.3%, *p* = 0.0459; Fisher’s Exact test). No other complication demonstrated statistically significant variation between the two groups. Anaemia remained the most frequently observed complication across both risk strata.

Long-term postoperative complications (>6 weeks) were primarily dominated by chronic hip pain, observed in 5.0% of patients. Some complications recorded in the early postoperative phase progressed into persistent issues at later follow-up. A single peri-prosthetic femoral fracture of the acetabulum (0.8%) occurred at eight months postoperatively following a fall. Radiographic assessments at all follow-up intervals demonstrated appropriate component positioning, with no evidence of acetabular cup migration, stem subsidence, or aseptic loosening. Furthermore, the dual mobility articulation exhibited no signs of polyethylene wear, component dissociation, or intra-prosthetic dislocation throughout the observation period. Late postoperative complications (>6 weeks) are listed in Table 9.

A comparison of long-term complications between fixation types is presented in Table 10. Long-term postoperative complications were observed in both the cemented and uncemented fixation groups, with no statistically significant differences identified across any category (all *p* > 0.05, Fisher’s exact test). The cemented group exhibited slightly higher rates of chronic hip pain (5.4% vs. 3.7%), trochanteric bursitis (2.2% vs. 0%), and leg length discrepancy (2.2% vs. 0%) compared to the uncemented group. Notably, no cases of sciatic nerve injury, deep infection, heterotopic ossification, or peri-prosthetic fracture were recorded in the uncemented cohort; however, the absolute incidence of these complications remained low in both groups, limiting definitive comparisons.

After merging ASA groups, no complications differed significantly (all *p* > 0.05, Fisher’s Exact test), though chronic hip pain was shown to be the most prominent long-term complications after DM-THA across all groups. Long-term complications by ASA grade are shown in Table 11.

### 3.5. Benchmarking Against National Outcomes

Thirty-day mortality in the study cohort was 0.8% (1/120), compared to 6.0% (4314/71,901) in the UK National Hip Fracture Database (NHFD 2024. A two-sample Z-test for proportions demonstrated a statistically significant difference between the two groups (*p* = 0.017). Thirty-day mortality rates are compared with NHFD benchmarks in Table 12.

## 4. Discussion

### 4.1. Intra-Prosthetic Dislocation

The complete absence of dislocations in our cohort underscores the elimination of classical dislocation, and importantly we also recorded no intra-prosthetic dislocations of the liner. Intra-prosthetic dislocation (IPD), did not occur in any case, reinforcing confidence in the implant’s locking mechanism. IPD of modern dual mobility liners is exceedingly rare (typically <1% incidence in the literature [20]), and our series mirrors this rarity. All radiographs, both immediate postoperative and at one-year follow-up, showed the components to be well-positioned and securely fixed, with no radiographic evidence of loosening. In a high-risk population where patients sustained femoral neck fractures (FNFs) trauma, traditionally prone to instability (dislocation rates of 4–10% are commonly cited for standard THA after FNF, with some series reporting rates as high as 22% [21,22]), achieving zero dislocations is noteworthy. Even the large HEALTH trial (NEJM 2019) found a 4.7% dislocation rate in the THA group (versus 2.4% for hemiarthroplasty), despite strict inclusion of healthier patients [23].

This finding aligns with a growing body of evidence that dual mobility implants dramatically reduce dislocation risk compared to conventional designs [8,22,24]. The dual articulation and enlarged effective head size (“head–neck ratio”) of the DM design increase the jump distance required for the head to dislocate [22], thereby resisting prosthetic dislocation even under extremes of motion and/or less-than-ideal positioning. For example, Zagorov et al. [25] found no dislocations with primary dual mobility THA in FNF patients, versus an 11.1% dislocation rate in those with a standard cup. Large registry data further support this stability benefit, noting about a 60% reduction in dislocation risk with dual mobility relative to fixed-bearing implants [26]. Similarly, a 2021 meta-analysis by Mufarrih et al. [27] observed an average dislocation rate of only ~1.9% with DM–THA for FNF, significantly lower than rates reported for single-bearing cups.

Notably, the surgical approach may also influenced dislocation outcomes: the posterior approach carries a higher baseline instability risk (~3–8% dislocation with a posterior approach [22] vs. 0.5–0.6% with lateral approach [28]). A recent UK multicentre study of fracture THA likewise reported a 0% dislocation rate with DM components versus 5.7% with conventional cups using a posterior approach [21], translating to over a fourfold reduction in dislocation risk without increasing other complications. At our institution, the posterior approach is primarily used. Importantly, we observed no compromise in function associated with this stability (as evidenced by the excellent postoperative scores discussed below) suggesting that any approach-related limp or muscle weakness was minimal.

Moreover, dual mobility technology may enable a more liberal rehabilitation. DM-THA has enhanced hip stability by increasing jump distance [29] and impingement-free range of motion [30], thereby reducing dislocation risk. These biomechanical advantages support earlier full weight-bearing and fewer motion restrictions, converting into better early stability through restored muscle tone and patient confidence in the new hip. Even typically high-risk patients (e.g., those with cognitive impairment or poor compliance) seem to benefit from the forgiving nature of dual mobility bearings. Prior studies have reported zero dislocations in dementia patients whose recovery is challenged because of difficulties in understanding and keeping the necessary hip precautions; thus, a more inherently stable implant leads to fewer complications when DM-THA is used for hip fractures [31]. 

### 4.2. Oxford Hip Score (OHS)

Beyond stability, functional recovery in this cohort has been excellent. At one-year follow-up, the mean Oxford Hip OHS was 41.0 ± 8.3, with 68.8% of patients achieving “excellent” outcomes (OHS ≥ 40. This corresponds to patients experiencing only mild residual symptoms in daily life. Such outcome are on a par with, if not exceeding, typical post-THA recovery for osteoarthritis patients [32]. For context, registry data indicate that the average OHS about three months after elective primary THA is ~34 points, improving to ~40 by one year [32]. Likewise Verhaegen et al. found that hip fracture patients treated with THA by arthroplasty specialists achieved a mean OHS of ~43 at final follow-up, statistically equivalent to the outcomes of matched THA patients treated for elective osteoarthritis [33]. In their study, the dislocation rate was 1.7% (with no dual mobility used), suggesting that when arthroplasty is performed under optimal conditions, fracture patients can attain functional scores on par with elective cases. Our OHS results, while slightly lower, remain within the “excellent” range and underscore the benefits of total hip arthroplasty in restoring function for displaced neck fractures.

Achieving an OHS in the 40 s so soon after surgery suggests that these hip fracture patients rapidly regained mobility and quality of life comparable to elective-surgery patients. Such robust patient-reported outcomes likely stem from multiple factors.

First, DM implants do not appear to compromise the range of motion or hip function; by design, they allow a large jump distance and arc of motion before impingement [34]. The greater range of safe motion and intrinsic stability may allow patients to move without fear, facilitating more aggressive rehabilitation and a return to activities. In our study, patients were mobilised as early as the first postoperative day under the supervision of physiotherapists, utilising the DM cup’s freedom of movement to encourage early functional use of the limb. This practice is consistent with reports that DM-THA can permit superior range of motion and functional scores versus conventional THA in elderly fracture patients [22]. Agarwala et al., for example, demonstrated significantly higher Harris Hip Scores and the ability to perform high-flexion activities (such as squatting or sitting cross-legged) in a dual mobility group compared to a standard THR group [22]. Mechanistically, the large effective head of a dual mobility implant delays impingement and permits a wider arc of motion before instability. This not only prevents dislocation but also enables patients to resume routine movements (like bending to dress or cutting toenails) with greater ease and confidence.

Second, the surgical approach and soft-tissue handling influence functional recovery. A lateral approach can risk abductor muscle impairment, but our centre primarily utilises a posterior approach, which generally has less impact on the hip abductors. This is confirmed by our high OHS results, implying that any limp or muscle weakness was minimal and transient. Indeed, dual mobility construct has been cited as an enabler for surgeons to use familiar approaches (like posterior) in fracture patients without incurring the usual penalty of higher dislocation rates [35,36].

In summary, the combination of implant stability and tailored surgical technique in our study allowed patients to achieve rapid and meaningful functional restoration after what is often a life-altering injury. These findings suggest that by minimising instability with a dual mobility cup, we attained both the low re-operation benefits of THA and excellent patient-reported function (mean OHS ~41) in an elderly fracture cohort.

### 4.3. Peri-Prosthetic Fractures (PPFs)

The incidence of peri-prosthetic femoral fracture in our study was low. Overall PPF occurred in 1.7% of patients, with early postoperative (≤90-day) PPF in only 0.8%. These rates are comparable to, or lower than, those reported in other series. Large registry-based studies of primary THA note that early postoperative PPF occurs in roughly 1–2% of cases [37].

Intraoperative or early postoperative femoral fractures are a known hazard in osteoporotic bone, particularly when uncemented stems or forceful impaction techniques are used [37]. For example, a recent high-volume single-centre report (6788 THAs) documented a 1.9% incidence of PPF within 90 days, with significantly more fractures occurring when a cementless “compaction” technique was used as opposed to traditional broaching (2.3% vs. 1.3%) [37]. Our low fracture rate possibly reflects careful surgical technique and appropriate implant choice for this vulnerable population. Most patients in our study received cemented femoral implants, mostly due to osteoporotic bone. In the context of hip fracture, adherence to cemented fixation has been emphasised to reduce PPF risk in osteoporotic bone [20]. Cementing the stem improves implant stability in poor-quality bone and is associated with lower PPF risks relative to press-fit designs, albeit at the cost of a small risk of cementation syndrome. Interestingly, in our study, both PPF events occurred in patients with cemented stems. One possible explanation is that patients who were deemed more suitable for cementless fixation were less fragile, with better bone quality, compared to the cemented population. While this is too few to draw firm conclusions, it suggests that factors like bone quality and fall dynamics, rather than the choice of cemented vs. uncemented stem, could be the primary determinants of these fractures.

Importantly, the dual mobility acetabular component itself has not been associated with any increase in PPF risk. A UK multicentre study observed a ~2% PPF rate overall with no difference between dual mobility and conventional bearing cohorts [21]. Likewise, our 0.8% early PPF incidence indicates that adopting the G7^®^ dual mobility cup (in conjunction with a cemented CPT stem) did not introduce new fracture complications compared to historical controls. Nevertheless, PPF remains a serious, albeit infrequent, complication, so vigilance is required to prevent and manage this issue. Measures such as gentle implant insertion techniques, appropriate patient selection (e.g., cemented stems for osteoporotic bone), and close postoperative monitoring are warranted to keep PPF rates low.

### 4.4. Early Complications

In our study, the most common early medical complication was postoperative anaemia (Hb < 70 g/L or 7 g/dL, and/or requires transfusion), which occurred in 6.6% of patients. This reflects perioperative blood loss combined with the limited physiological reserve of frail, elderly traumatic fracture patients. While our transfusion rate is relatively modest (transfusion rates up to 22.2% have been reported after hip arthroplasty in some centres [38]), it nevertheless underlines the need for proactive management. We did not observe a significant difference in anaemia incidence between low-risk (ASA I–II) and high-risk (ASA III–IV) patients, a finding that runs counter to the general expectation that higher ASA class predicts greater transfusion requirements and medical complications [39]. This lack of disparity may be due to effective perioperative protocols in our hospital or the limited sample in each subgroup. In any case, the occurrence of anaemia in roughly one in fifteen patients highlights the importance of vigilant blood conservation strategies. Preoperative optimisation (for example, treating pre-existing anaemia), meticulous surgical haemostasis, and a low threshold for postoperative transfusion in high-risk patients are all prudent measures to minimise the impact of perioperative blood loss.

Venous thromboembolism (VTE) was another significant early complication, with an incidence of 4.2% in our cohort. Encouragingly, no patient suffered a fatal pulmonary embolism (PE). Most cases of deep vein thrombosis (DVT) and PE were observed in the ASA III/IV cohort, which is consistent with arthroplasty literature showing that patients with greater comorbidity burdens (higher ASA scores) have elevated VTE risk after hip or knee replacement [40]. In the context of FNF, several factors compound the thromboembolic risk: advanced age, the trauma of fracture, unavoidable preoperative immobilisation, and common comorbidities (e.g., cardiac failure, malignancy, or coagulopathies). Our VTE rate is slightly higher than those reported in elective primary THA series with rigorous prophylaxis, which is understandable given the acute fracture setting. This highlights the importance of vigilance during the perioperative period and taking proactive steps such as starting anticoagulants early, using compression devices, and making patients move as soon as possible after surgery. This underscores the need for heightened vigilance in the perioperative period and aggressive thromboprophylaxis in fracture patients, including early initiation of chemical prophylaxis, use of mechanical compression devices, and early postoperative mobilisation. Identifying ASA III-IV patients as particularly at risk allows clinicians to tailor prophylactic strategies (for instance, extended anticoagulation post-discharge and closer monitoring) to mitigate VTE in this vulnerable group.

### 4.5. Long-Term Complications

At one-year follow-up, chronic hip pain was reported by 5.0% of our patients. This proportion is relatively low compared to some reports in the literature, for example, a large registry-based study from Denmark found that about 12% of THA patients experienced significant chronic hip pain one-year postoperatively [41]. In our cohort, an intriguing (though not statistically significant) observation was that patients in the lower ASA classes (I-II) reported a slightly higher rate of persistent hip pain than those in ASA III-IV. This counterintuitive trend contradicts the expectation that patients with more comorbidities (higher ASA) would have worse pain outcomes [42]. One possible explanation is that healthier, more active patients have higher functional expectations and may be more sensitive to residual pain, whereas those with greater frailty may have lower activity levels or different pain perceptions. Regardless, these findings have important implications for rehabilitation: even patients who are relatively healthy at baseline may benefit from targeted pain management and physical therapy to address lingering pain after fracture THA. Early identification and treatment of chronic pain, along with setting appropriate expectations, could further optimise recovery [43]. Overall, the low incidence of long-term pain in our series is encouraging, and it suggests that most patients regain a pain-free status comparable to elective THA populations. Ongoing follow-up will be important to monitor whether any new late-onset issues (such as heterotopic ossification or polyethylene wear-related symptoms) arise in subsequent years.

### 4.6. Mortality Rate and Benchmarking

Our 30-day mortality was 0.8%, which is substantially lower than expected for this patient group. By comparison, the UK National Hip Fracture Database (NHFD) reports an approximately 6.0% risk-adjusted 30-day mortality for hip fracture patients [44,45]. Notably, performing a THA for a fracture is a more extensive procedure than the standard hemiarthroplasty, raising concerns that it could increase short-term mortality in frail patients. However, our results indicate that with optimised perioperative care, even vulnerable older patients can safely tolerate the bigger operation. We acknowledge that patient selection likely contributed to our low mortality: candidates chosen for THA (as opposed to hemiarthroplasty) tend to be healthier (e.g., better cognitive and ambulatory status), which inherently confers a survival advantage.

Even so, our 0.8% 30-day mortality is strikingly low and suggests that our multidisciplinary care pathway effectively mitigated many drivers of hip fracture mortality. In our institution, nearly all fracture patients receive surgery within 24 h of presentation, often after proactive optimisation by a geriatric medicine team (addressing issues like anaemia, hydration, and nutrition preoperatively). We also employ an enhanced recovery programme—including regional anaesthesia for pain control, early mobilisation (often on day one post-op), and close medical monitoring—to reduce perioperative risk. These measures likely contributed to the encouraging survival outcome in our cohort. This orthogeriatric co-management model has demonstrated improvements in survival and early outcomes in many other studies [46].

Reassuringly, the use of a dual mobility implant itself does not appear to negatively impact mortality. Prior studies have found no significant difference in early- or medium-term mortality between hip fracture patients treated with dual mobility cups versus those receiving standard implants [21]. Thus, our experience suggests that adopting a dual mobility THA approach in high-risk fracture patients is feasible without incurring the higher early mortality historically associated with this population. In summary, through a combination of careful patient selection, expedited surgery, optimised medical management, and the enhanced stability of the dual mobility implant, we achieved a 30-day mortality below the national averages for hip fracture care in the UK.

### 4.7. Limitation of the Study

Despite the strengths of our study design and results, several limitations must be considered.

Single-centre design: This investigation was conducted at a single institution, which inherently limits the external validity of the findings. Outcomes from one centre may not fully apply to other hospitals or patient populations, as differences in patient demographics, surgical techniques, and perioperative protocols could lead to different results. Therefore, exercise caution when extrapolating our data to broader settings.

Short-to-mid-term follow-up: Our follow-up was limited to the short-to-midterm, so longer-term outcomes remain unknown. We could not assess the durability of the G7^®^ dual mobility implant or detect complications that might only manifest in the long run. The absence of long-term data introduces uncertainty about the sustained benefits of this implant and the possibility of late complications or failures (for example, polyethylene wear or very late dislocations) that could emerge over time. Any conclusions regarding implant longevity or late complication rates must therefore be considered preliminary.

Limited subgroup sample size: Only a few patients in our cohort received uncemented femoral stems, which limits the power to compare outcomes between cemented and uncemented subgroups. Such small numbers increase the risk of a Type II error (i.e., missing a true effect) and warrant caution in interpreting an apparent lack of difference. As a result, findings related to these sub-cohorts (for example, the performance of uncemented stems in this context) should be viewed as exploratory rather than definitive.

Statistical consideration: The absence of a post hoc power analysis represents a methodological limitation. As this study was retrospective and exploratory, the sample size was determined by consecutive case inclusion rather than a priori power calculation. Consequently, while the study’s findings are informative, they should be interpreted as descriptive and hypothesis-generating rather than confirmatory.

A further limitation is the absence of precise records regarding the surgical approach used in each case. While the posterior approach predominated, some patients were treated with anterolateral or direct lateral exposures. Although this introduces procedural heterogeneity, consistent use of the same implant system, perioperative care protocols, and follow-up criteria helps mitigate the impact on outcome interpretation. Future prospective studies should document approach-specific outcomes to clarify any potential influence of surgical exposure on stability or complication rates.

Lack of blinding: We did not employ blinding for patients, surgeons, or outcome assessors, introducing potential bias. Knowledge of the implant type by the care team and patients could influence postoperative management or subjective outcome reporting (performance and response bias). Similarly, assessors aware of treatment allocation might have unconscious expectations that skew evaluations. Without blinding, there is an inherent risk that positive outcomes were overestimated or certain negative outcomes under-recognised. Thus, our results must be interpreted with appropriate caution regarding possible bias.

Potential confounding from patient selection: Finally, as a non-randomised observational study, our findings are subject to selection bias and unmeasured confounders. Patients were not randomly assigned to treatments. Inclusion was based on clinical decisions and eligibility criteria that may have favoured enrolling healthier or more active individuals for DM-THA. If those selected patients inherently had better prognoses, it could confound the relationship between the dual mobility implant and the outcomes seen. While we accounted for known variables, it is possible that some confounding factors still influenced the results.

### 4.8. Future Directions

Long-Term Outcomes: Extended follow-up studies are needed to determine the long-term durability and performance of the G7^®^ dual mobility construct in fracture patients. Our evidence is currently limited to short-term results, so the sustained benefits of dual mobility, for example, whether the low dislocation rates continue over many years, remain unproven. Additionally, any late complications such as polyethylene wear, component loosening, or very late peri-prosthetic fractures are still unknown. Long-term surveillance (5–10+ years) of this cohort will be needed to confirm that the early advantages of the dual mobility implant persist over time.

Randomised Trials and Comparative Studies: High-quality comparative research is essential to validate the benefits of dual mobility THA in femoral neck fractures. A sufficiently powered randomised controlled trial (RCT) with dislocation as a primary endpoint would definitively quantify the reduction in instability provided by dual mobility components relative to standard THA or hemiarthroplasty. In addition to dislocation rates, such trials should evaluate functional recovery, complication rates, and mortality to ensure that the stability gains of dual mobility do not come at the expense of other outcomes. (Notably, some meta-analyses have suggested worse certain short-term outcomes with dual mobility in fractures [47], underlining the importance of rigorous head-to-head data). Robust comparative evidence will help surgeons determine the optimal arthroplasty approach for displaced FNFs.

Optimal Implant Fixation Strategies: Further investigations should identify the best fixation methods and surgical techniques when using dual mobility THA in fragility fracture patients. Elderly fracture populations often have poor bone quality, which raises the question of cemented versus uncemented fixation for both the acetabular cup and femoral stem in the context of dual mobility. Our data unexpectedly showed no significant difference in early outcomes (dislocations, complications, or PPF) between cemented and uncemented femoral stems, contradicting some early reports. Future research should explore whether these findings hold in larger samples and different settings, and whether cementation of the dual mobility cup offers any advantage or disadvantage. Defining optimal fixation strategies (for example, fully cemented vs. hybrid or cementless approaches) in the context of dual mobility will help improve surgical protocols for this technology.

Health-Economic Evaluation: The broader health-economic implications of adopting dual mobility THA for hip fractures should be considered alongside clinical outcomes. Dual mobility implants are more expensive up front than standard femoral heads or liners, but our evidence and others suggest this initial cost can be offset by avoiding downstream complications. Even a single dislocation in an elderly patient incurs significant costs: hospital readmission, revision surgery, rehabilitation, and the morbidity of prolonged immobility; so, maintaining a zero-dislocation rate in our series likely prevented several such costly events. Health-economic models support this trade-off: a Markov analysis based on registry data estimated that routine use of dual mobility in primary THA could save approximately EUR 28 million per 100,000 cases by preventing dislocations and their sequelae [26]. Barlow et al. reported that a dual mobility construct would remain cost-effective even if priced up to about USD 1000 more than a conventional implant, given the high expense of treating dislocations [48]. Moreover, improvements in implant design have mitigated earlier issues like wear or intra-prosthetic dislocation [22], contributing to excellent longevity (for instance, Neri et al. documented 25-year survivorship with no dislocations using a dual mobility cup [49]). These advances bode well for the long-term cost-effectiveness of dual mobility THA: by providing superior early stability and function, the strategy likely reduces downstream healthcare utilisation, meaning any initial implant cost premium is justified by lower complication-related expenditures over time. Nonetheless, formal health-economic evaluation of dual mobility THA in fracture patients is warranted. A recent modelling study suggests that using dual mobility components for displaced FNFs can be cost-effective in patients under 80 years old if the dislocation risk is substantially reduced [13]; however, such analyses rely on assumptions that must be validated in real-world practice. Future studies should incorporate rigorous economic endpoints (e.g., quality-adjusted life years, index hospitalisation and implant costs, costs of complications or revisions, and overall health system utilisation) alongside clinical outcomes. If dual mobility THA indeed lowers revision rates or the need for prolonged institutional care by reducing complications, it could offer significant long-term cost savings despite the higher implant price. Confirming this through prospective cost-effectiveness studies or large registry analyses will be important for policymakers and hospital administrators when weighing the broader adoption of dual mobility implants in hip fracture care.

## 5. Conclusions

Our early experience at our centre with dual mobility total hip arthroplasty (DM-THA) for femoral neck fractures (using the third-generation Zimmer Biomet G7 system) has been highly encouraging. In our series, no dislocations occurred, the 30-day mortality was 0.8% (well below national averages), patient-reported functional outcomes were excellent (mean Oxford Hip Score of 41, comparable to outcomes of elective THA), and the overall complication rate was low. Together, these findings support the growing consensus that DM-THA is an effective strategy for displaced FNFs in elderly or high-risk patients, offering the benefits of a total hip replacement (improved mobility) while minimising the historically high risk of dislocation. These favourable outcomes likely reflect a combination of factors, including advanced implant design (providing intrinsic stability through the dual mobility cup), meticulous surgical technique (appropriate approach with careful soft-tissue handling), careful patient selection and optimisation (relatively fit patients optimised for surgery), and comprehensive multidisciplinary care (e.g., geriatric co-management). Consistent with other recent studies, our findings suggest that the dual mobility approach addresses the key vulnerabilities of this fracture population, namely instability and limited mobility, without increasing operative risk. While continued long-term follow-up is required to confirm that these early gains are sustained, DM-THA holds considerable promise for optimising stability, function, and overall recovery in the management of FNFs.

## Figures and Tables

**Table 1:** Demographics and clinical characteristics.

Characteristic	Value	%
Age Distribution		
Mean age ± SD (years)	71.6 ± 9.4 ^1^	-
Range (years)	43–92	-
Gender Distribution		
Female	89	74.2%
Male	31	25.8%
Total patients	120	100%
ASA Scores ^2^		
ASA I	5	4.2%
ASA II	74	61.7%
ASA III	40	33.3%
ASA IV	1	0.83%
Total patients	120	100% ^3^

^1^: Values are presented as mean ± standard deviation (SD); range indicates minimum maximum. ^2^: ASA = American Society of Anaesthesiologists physical status classification. ^3^: Percentages are based on the total number of patients (n = 120) and may not sum up to 100% due to rounding.

**Table 2:** Stem type distribution in Safety and Functional Cohorts.

Stem Type	Subtype ^1^	Safety Cohort (n = 120)	Functional Cohort (n = 96)
Cemented	CPT	91 (75.8%)	72 (75.0%)
	C-Stem	2 (1.7%)	1 (1.0%)
Total Cemented	-	93 (77.5%)	73 (76.0%)
Uncemented	Corail	24 (20.0%)	20 (20.8%)
Unknown ^2^	-	3 (2.5%)	3 (3.1%)
Total	-	120 (100%)	96 (100%)

^1^: CPT: collared polished tapered stem; C-Stem: modular cemented stem; and Corail: uncemented hydroxyapatite-coated stem. ^2^: Unknown type of stem due to unclear operative notes, excluded from stem comparisons.

**Table 3:** Dislocation incidents at one-year follow-up.

Dislocation	Count	%
Cemented	0	0.0%
Uncemented	0	0.0%
Total	0	0.0%

**Table 4:** Mean Oxford Hip Score at one-year follow-up.

Fixation Type	Mean OHS	SD	Range	95% Confidence Interval for Mean	
				Lower Bound	Upper Bound
Cemented	40.2	8.9	5–48	38.1	42.3
Uncemented	43.9	4.5	34–38	41.8	46.0
Total	41.0	8.3	5–48	38.3	42.7

Comparison between cemented and uncemented groups performed using the Mann–Whitney U test.

**Table 5:** Peri-prosthetic fracture (PPF) of the acetabular or femoral implant at one-year follow-up.

Fixation Type	PPF Count	%
Cemented	1 ^1^	1.0%
Uncemented	0	0.0%
Total	1	1.0%

^1^: One PPF of the acetabulum occurred in the cemented fixation group.

**Table 6:** Early postoperative complications (≤6 weeks).

Complication	Count	Percentage (%)
Pulmonary Embolism/Deep Vein Thrombosis	5	4.2%
Limb Length Discrepancy	1	0.8%
Trochanteric Bursitis	1	0.8%
Acute Kidney Injury (AKI)	3	2.5%
Trapped Sciatic Nerve	1	0.8%
Anaemia	8	6.7%
Hypotension	3	2.5%
Lower Respiratory Tract Infection (LRTI)	1	0.8%
Peri-prosthetic Fracture	1	0.8%
Surgical Site Infection	3	2.5%
Blisters	1	0.8%
Atrial Fibrillation (AF)	1	0.8%
Sepsis	1	0.8%

**Table 7:** Early postoperative complications by fixation type.

Complication	Cemented n = 93 (Count, %)	Uncemented n = 24 (Count, %)
PE/DVT	4 (4.3%)	2 (8.3%)
Limb Length Discrepancy	1 (1.1%)	0 (0.0%)
Trochanteric Bursitis	1 (1.1%)	0 (0.0%)
Acute Kidney Injury (AKI)	2 (2.2%)	1 (4.2%)
Trapped Sciatic Nerve	1 (1.1%)	0 (0.0%)
Anaemia	7 (7.5%)	1 (4.2%)
Hypotension	2 (2.2%)	1 (4.2%)
Lower Respiratory Tract Infection	0 (0.0%)	1 (4.2%)
Peri-prosthetic Fracture	0 (0.0%)	1 (4.2%)
Surgical Site Infection	2 (2.2%)	0 (0.0%)
Blisters	1 (1.1%)	0 (0.0%)
Atrial Fibrillation (AF)	1 (1.1%)	0 (0.0%)
Sepsis	1 (1.1%)	0 (0.0%)

Fisher’s Exact test: All *p* > 0.05—no significant difference in complications between the fixation groups.

**Table 8:** Early postoperative complications by ASA scores.

Complication	ASA I + II n = 79 (Count, %)	ASA III + IV n = 41 (Count, %)
PE/DVT ^1^	1 (1.3%)	4 (9.8%)
Limb Length Discrepancy	0 (0.0%)	1 (2.4%)
Trochanteric Bursitis	0 (0.0%)	1 (2.4%)
AKI	2 (2.5%)	1 (2.4%)
Trapped Sciatic Nerve	1 (1.3%)	0 (0.0%)
Anaemia	4 (5.1%)	4 (4.9%)
Hypotension	1 (1.3%)	2 (1.7%)
LRTI	0 (0.0%)	1 (2.4%)
Peri-prosthetic Fracture	1 (1.3%)	0 (0.0%)
Surgical Site Infection	1 (1.3%)	1 (2.4%)
Blisters	0 (0.0%)	1 (2.4%)
Atrial Fibrillation (AF)	0 (0.0%)	1 (2.4%)
Sepsis	0 (0.0%)	1 (2.4%)

^1^: Fisher’s Exact test for PE/DVT: *p* = 0.0459—significantly higher in high-risk group. Other complications’ Fisher’s Exact test: *p* > 0.05—no significant difference in complications between the ASA groups.

**Table 9:** Long-term postoperative complications (>6 weeks).

Complication	Count	Percentage (%)
Chronic Hip Pain	6	5.0%
Leg Length Discrepancy	2	1.7%
Trochanteric Bursitis	2	1.7%
Peri-prosthetic Fracture	1	0.8%
Sciatic Nerve Injury	1	0.8%
Deep Infection	1	0.8%
Heterotopic Ossification	1	0.8%
Complication	Count	Percentage (%)
Chronic Hip Pain	6	5.0%
Leg Length Discrepancy	2	1.7%
Trochanteric Bursitis	2	1.7%
Peri-prosthetic Fracture	1	0.8%
Sciatic Nerve Injury	1	0.8%
Deep Infection	1	0.8%
Heterotopic Ossification	1	0.8%

**Table 10:** Long-term postoperative complications by fixation type.

Complication	Cemented n = 93 (Count, %)	Uncemented n = 24 (Count, %)
Chronic Hip Pain	5 (5.4%)	1 (0.8%)
Leg Length Discrepancy	2 (2.2%)	0 (0.0%)
Trochanteric Bursitis	2 (2.2%)	0 (0.0%)
Peri-prosthetic Fracture	1 (1.1%)	0 (0.0%)
Sciatic Nerve Injury	1 (1.1%)	0 (0.0%)
Deep Infection	1 (1.1%)	0 (0.0%)
Heterotopic Ossification	1 (1.1%)	0 (0.0%)
DVT	0 (0.0%)	0 (0.0%)

Fisher’s Exact test: all *p* > 0.05—no significant difference in complications between the fixation groups.

**Table 11:** Long-term postoperative complications by ASA scores.

Complication	ASA I + II n = 79 (Count, %)	ASA III + IV n = 41 (Count, %)
Chronic Hip Pain	5 (6.3%)	1 (2.4%)
Leg Length Discrepancy	2 (2.5%)	0 (0.0%)
Trochanteric Bursitis	1 (1.3%)	1 (2.4%)
Peri-prosthetic Fracture	0 (0.0%)	1 (2.4%)
Sciatic Nerve Injury	1 (1.3%)	0 (0.0%)
Deep Infection	0 (0.0%)	1 (2.4%)
Heterotopic Ossification	1 (1.3%)	0 (0.0%)

Fisher’s Exact test: all *p* > 0.05—no significant difference in complications between the fixation groups.

**Table 12:** Thirty-day mortality comparison.

Source	Mortality Count	%
This Study	1/120	0.8%
NHFD 2024	4314/71,901	6.0%

Z-test *p* = 0.017: This study has significantly lower mortality rate than the NHFD 2024.

## Data Availability

Data supporting reported results can be provided on request, welcome to send requesting emails to the corresponding authors.

## Abbreviations

The following abbreviations are used in this manuscript:

FNF	Femoral Neck Fracture
THA	Total Hip Arthroplasty
DM	Dual Mobility
PPF	Peri-Prosthetic Fracture
DM-THA	Dual Mobility Total Hip Arthroplasty
OHS	Oxford Hip Score
NHFD	National Hip Fracture Database
AP	Anterior–Posterior
DVT	Deep Vein Thrombosis
VTE	Venous Thromboembolism
PE	Pulmonary Embolism
QALY	Quality-Adjusted Life Years
AKI	Acute Kidney Injury
LRTI	Lower Respiratory Tract Infection
AF	Atrial Fibrillation
